# The Course of the V2 Segment of the Vertebral Arteries in Klippel-Feil Syndrome: A Case Report

**DOI:** 10.7759/cureus.3038

**Published:** 2018-07-24

**Authors:** Asad Rizvi, Joe Iwanaga, Rod J Oskouian, Marios Loukas, R. Shane Tubbs

**Affiliations:** 1 Medicine, St. Georges University School of Medicine, St. Georges, GRD; 2 Medical Education and Simulation, Seattle Science Foundation, Seattle, WA, USA; 3 Neurosurgery, Swedish Neuroscience Institute, Seattle, WA, USA; 4 Anatomical Sciences, St. George's University, St. Georges, GRD; 5 Neurosurgery, Seattle Science Foundation, Seattle, WA, USA

**Keywords:** klippel-feil syndrome, vertebral artery, ct angiography, imaging, cervical vertebrae, surgical management

## Abstract

Klippel-Feil syndrome is a congenital disorder characterized by the fusion of one or more cervical vertebrae leading to limitations in the rotation, extension, and flexion of the neck and possible neurological symptoms. Other abnormalities can also be found in these patients. The anatomy of the vasculature can be abnormal in these patients including variations in the course and origin of the vertebral arteries potentially leading to intra-operative complications. Herein, we report a case of Klippel-Feil syndrome and detail the course of the vertebral arteries in an osteological specimen.

## Introduction

Klippel-Feil syndrome is characterized by the fusion of one or more cervical vertebrae [1]. Klippel and Feil [2] first identified this condition in a 46-year-old tailor having a short neck with a limited range of motion and a low posterior hairline. This condition can be inherited in autosomal dominant and recessive patterns, and many classification schemes have been proposed. Patients usually present with neurological symptoms, pain, and limited rotation, flexion, and extension of the neck. The age of presentation depends on the degree of severity of the fusion and other abnormalities, but these patients usually present at a later age [1,3]. Radiographs are essential in the evaluation of these patients to assess the degree of cervical fusion and other associated abnormalities [4]. Many management options exist depending on the symptoms and severity of the condition including modification of activities, bracing, arthrodesis, and placement of screws and plates [1].

The course of the vertebral arteries in Klippel-Feil syndrome is important, as an anomalous artery can be injured during surgical screw placement. The literature describes several vascular abnormalities associated with Klippel-Feil syndrome [5-8] including variations in the course and origin of the vertebral arteries [9-12]. Herein, we report a case of Klippel-Feil syndrome and detail an unusual course of the vertebral arteries in a bone specimen.

## Case presentation

**Figure 1:**
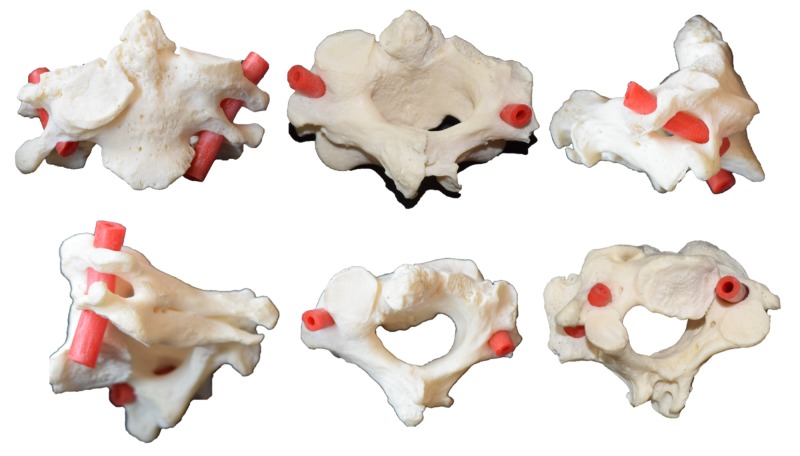
First row: left, anterior view; middle, posterior view; right, right lateral view. Second row: left, left lateral view; middle, superior view; right, inferior view. For all images, note the varied course of the V2 segment of the vertebral artery as demonstrated by the red tubing.

Fusion of the C2 and C3 vertebrae was identified in a skeletal sample of an adult male (Figure 1). No other bony variations, such as cervical ribs, were noted in the specimen. In this specimen, irregularity of the right-sided superior articular facet surface and a short odontoid process and anomalous articular facet on the left side of the lateral odontoid process, characteristic of Klippel-Feil syndrome, were noted as well The vertebral canal between C2 and C3 was stenotic, and the spinous processes of these vertebrae were bifid. There was a circumferential fusion between the two vertebrae. Upon passing red tubing through the adjacent transverse foramina of the left and right sides, the course of the vertebral arteries was determined to be very asymmetric (Figure 1). In the sagittal plane, the left vertebral artery traveled almost vertically but was angled posteriorly by about 10 degrees. However, the right vertebral artery at this vertebral level traveled more horizontally and was tilted posteriorly by about 45 degrees. Using the odontoid process as the midline, the left vertebral artery was found to be 2.8 cm from the midline and the right vertebral artery 2.5 cm from the midline.

## Discussion

Embryology of the cervical vertebrae and the vertebral artery

The vertebrae develop from the paraxial mesoderm formed by the process of gastrulation beginning on day 14 after conception. The paraxial mesoderm subdivides into smaller structures called somites through the process of segmentation between days 20 and 30. Somites further divide into sclerotomes (later form the vertebrae), myotomes, and dermatomes. A re-segmentation process occurs when the caudal part of the sclerotome of one somite fuses with the rostral part of the other, forming the vertebral body. Klippel-Feil syndrome is believed to result from mutations in the genes involved in regulating the segmentation and re-segmentation processes, and reports in the literature have identified several candidate genes [1].

The formation of the vascular system and the above-described development of the vertebrae occur simultaneously. During days 21 to 36 of gestation, six pairs of aortic arches are formed. The fourth right aortic arch forms the proximal part of the right subclavian artery between days 32 and 36, and the left fourth aortic arch persists to form a part of the adult aortic arch. During days 21 to 29, 30 pairs of dorsal intersegmental arteries arise from the dorsal aorta to supply the somites, and the neural tube and the first nine pairs anastomose with one another, forming the vertebral arteries. The proximal parts of the first six intersegmental arteries regress with the descent of the heart, leading to the vertebral arteries arising from the subclavian artery in adults. Finally, the seventh intersegmental artery forms the left and most of the right subclavian arteries. Bavinck et al. proposed that interruption in the blood supply through the vertebral arteries during embryonic development leads to Klippel-Feil syndrome and the associated vascular anomalies [13].

Klippel-Feil syndrome and the course of the vertebral artery

The usual course of the vertebral arteries can be divided into four parts: The first or the pre-foraminal part (V1) begins as the vertebral artery originates from the proximal subclavian artery and courses vertically upward to enter the transverse foramen of C6. In the second or the foraminal part (V2), it passes through the transverse foramen of C6-C1. The third or the extradural part (V3) courses on the upper surface of the posterior arch of the atlas (groove for vertebral arteries) and then enters the foramen magnum. The fourth or the intradural part (V4) is when the artery enters the dura mater [14].

Reports of variations in the course and origin of the vertebral arteries associated with Klippel-Feil syndrome include a case of a 15-year-old woman with the right vertebral artery originating from the carotid artery and then ascending vertically without entering the transverse foramina. Additionally, the left vertebral artery was hypoplastic in this patient [9]. Futane et al. [10] reported a small caliber right vertebral artery and a persistent left first intersegmental artery in a 26-year-old man with Klippel-Feil syndrome. Tian et al. [11] described a case of a 12-year-old female patient with Klippel-Feil syndrome with a high-riding right vertebral artery at C2. Another report [12] described a sinuous course of the V3 segment between C1 and C2 due to the ponticulus posticus of C2 in a 68-year-old woman with Klippel-Feil syndrome.

The management of patients with Klippel-Feil syndrome depends on the severity of the condition and can involve surgical screw placement. The vertebral artery is at risk of injury during such procedures [15-16]. Therefore, pre-operative imaging of the craniocervical junction with, for example, computed tomography (CT) angiography is essential for identifying the course of the vertebral arteries to avoid intra-operative complications [10,17].

## Conclusions

The literature describes some variations in the course and origin of the vertebral arteries in Klippel-Feil syndrome. These reports underscore the importance of pre-surgical or intraoperative imaging to identify any anomalies in the vertebral arteries and the need to exercise prudence during surgery to avoid potential complications. The case presented here offers a unique window into the anomalous course of the vertebral arteries in a skeletal specimen of an adult male with Klippel-Feil syndrome.
